# Trace Elements and Contaminants Concentrations in Tissues of Caspian Seals (*Pusa caspica*) along the Iranian Coast

**DOI:** 10.3390/toxics11010039

**Published:** 2022-12-30

**Authors:** Seyedeh Malihe Hoseini, Somayeh Namroodi, Amir Sayadshirazi, Annalisa Zaccaroni

**Affiliations:** 1 Department of Environmental Sciences, Faculty of Fisheries and Environmental Sciences, Gorgan University of Agricultural Sciences & Natural Resources, Gorgan 4913815739, Iran; 2 Caspian Seal Conservation Center, Ashooradeh Island, Iran; 3 Department Veterinary Medical Sciences, University of Bologna, 47042 Cesenatico, Italy; 4 MarLab, 06250 Mougins, France

**Keywords:** endangered species, *Pusa caspica*, trace elements, organochlorine pesticides, organophosphorus pesticides

## Abstract

The Caspian seal (*Pusa caspica*) is an endangered species that only lives in the Caspian Sea. Little information is available on its exposure to contaminants, and no data exists for Southern sub-populations. From 2011 to 2016, tissues samples were collected from 20 Caspian seals to (i) Define the concentration of trace elements in five different matrices and the concentration of 30 pesticides in their blubber; (ii) Determine whether differences in contaminant concentrations are age- or sex-related; (iii) Evaluate if detected concentrations can represent a risk to the species. Age- and sex-related variations were detected for Zn and Hg in the blubber and Fe in the kidney by age only. Exceptionally high Hg concentrations and low levels of hepatic Zn were detected, raising some concern about the reproductive health of seals. Similarly, the DDTs levels detected were in the range of adverse reproductive effects in marine mammals. Based on these results, potentially adverse effects on the immune and endocrine systems of the Caspian seal cannot be ruled out. Therefore, it is of the utmost importance that pollutant monitoring becomes an integral component of conservation strategies for the Caspian seal.

## 1. Introduction

The Caspian Sea is the largest land-locked saltwater lake in the world. Azerbaijan, Kazakhstan, Turkmenistan, Iran, and Russia surround it. One hundred and thirty inlet rivers and no outlets characterize it, leading to a continuous increase in organic and inorganic substances from agricultural and industrial areas, which accumulate in the seawater and sediments [1,2,3]. Among the different sources of pollutants—trace elements, organochlorine, and organophosphorus pesticides- the rivers flowing into the northern basin of the Caspian and the Absheron Peninsula, Azerbaijan, are significant [3]. Trace elements are contaminants whose concentration increases with time in the Caspian waters due to industrial and urban discharges and draining from the mining and industrial regions of Georgia and Armenia [1,2,4,5,6,7,8]. Trace elements are environmentally stable, do not degrade through bacterial metabolic pathways such as organic pollutants, and undergo bioaccumulation in the food web. Above certain thresholds, they become toxic [9,10,11,12,13,14] and cause adverse health effects [i.e., immune-, geno-, cytotoxic effects, reproduction impairment, and endocrine alterations [12,15] to long-lived, top marine predators [16,17,18,19,20,21,22,23,24,25,26].

Due to the increasing demand for agricultural practices, pesticide use has dramatically increased in recent decades. Intense agricultural activity in northern coastal areas of Iran has led to high pesticide usage, contributing to the Caspian Sea’s organic contamination by leaching and runoff into rivers [2,27,28,29,30,31]. Because of the chronic adverse effects of organochlorine pesticides (OCs) on animals and humans, their use has been banned since late 1970 and early 1980; since then, the use of organophosphorus pesticides (OPs), which are more easily degraded in nature—although they present a particular ability of bioaccumulation in the food chain and show long term adverse effects on the living organism—has increased [32,33,34,35]. By the late 1980s and early 1990s, immune impairment, reproductive failure, and viral epizootics (among others) had been associated with elevated concentrations of tissue residues compared with concentrations from healthy animals in aquatic top predators such as Baltic (*Pusa hispida botnica*) and Weddell (*Leptonychotes weddellii*) seals, striped dolphins (*Stenella coeruleoalba*), Atlantic bottlenose dolphin (*Tursiops truncatus*), and Caspian seals (*Pusa caspica*) [36,37,38,39].

The Caspian seal is a relict phocine species, taxonomically related to ringed (*Pusa hispida*) and Baikal (*Pusa sibirica*) seals; it breeds on ice in the northern part of the Caspian Sea and then spreads all over the Sea during the non-breeding season. It is considered a top predator of the Sea, and it feeds mainly on common Caspian kilka (*Clupeonella* sp.), Caspian silverside (*Atherina mochin*), Caspian gobies (Gobiidae), and crustaceans. Its actual population size ranges from 104000 to 168000 individuals, with an overall relative decline in a three-generation period (1955–2015) of 70%, classifying this species as endangered by IUCN [40]. Among the threats to this species, exposure to contaminants should also be considered, with organic pollutants representing the most monitored chemicals. Organic compounds and trace elements are not considered high enough to induce adverse effects, as Goodman et al. [40] stated in the latest IUCN species assessment. However, it should be noted that the only existing data refer to the 1997–2001 canine distemper virus (CDV) epizootic and the northern seals sub-population [16,26,39,41,42,43,44,45,46,47], while little is known on contamination along Iranian coasts, even though sea currents transport entrapped pollutants, discharged locally or from adjacent Azerbaijan and even Russia, along the Iranian coast [6]. After the outbreak’s end, no further follow-up of trace elements and pesticide effects on the health status of and surveys on pesticide bioaccumulation in Caspian seals were performed [42]. However, these contaminants’ monitoring studies on Caspian Sea pollution have been continuously performed on other Caspian species since 2001 [27,28,29,30,48,49,50,51].

This study aimed to: (i) Define the concentration of trace elements in five different matrices, (ii) Define the concentration of pesticides (15 OCs and 15 OPs) in blubber; (iii) Determine whether differences in contaminant concentrations are age- or sex-related; (iv) Evaluate if detected concentrations can represent a risk to the species.

## 2. Materials and Methods

### 2.1. Ethical Aspects

This study has been performed after approval by the Ethics Committee of the Deputy of Natural Environment of Golestan Province (Permit Number 125/7894, 15 September 2016). For the study, only dead, by-catched, net-entangled seals were considered; all carcasses were assigned a condition code 2. Consequently, no seal was explicitly euthanized for this study or for any other reason.

### 2.2. Sampling

**Figure 1:**
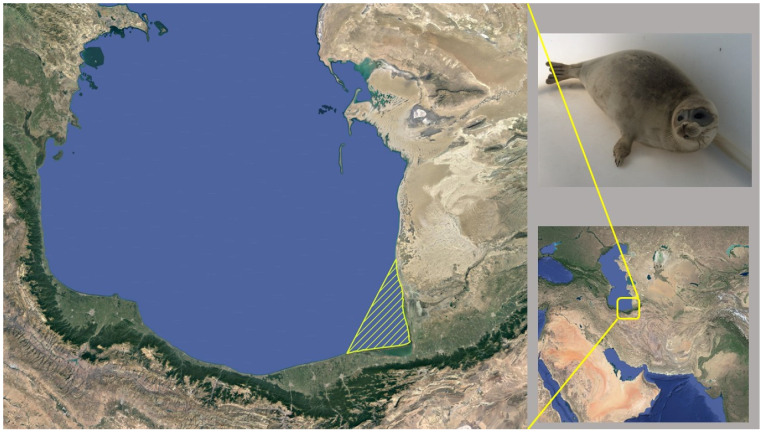
Sampling area (yellow-stripped area on the left) of stranded Caspian seals in Iran (2013–2016).

Tissue samples from the blubber (B), liver (L), kidney (K), heart (H), and skeletal muscle (M) were collected from 20 stranded Caspian seals (10 males, 10 females) from Bandar Torkaman and Miankaleh peninsula, Southern Caspian Sea, North-East Iran, in 2013–2016 (Figure 1).

Sampling was performed at the Gorgan University of Agricultural Sciences and Natural Resources. Before sampling, morphological parameters (straight length SL, axillary girth AG, and blubber thickness BT) were registered, and the age of the seals was determined after Amano et al. [52]. Each seal was assigned to the juvenile or adult age class, setting the threshold age between juveniles and adults at 8 years, following what was reported in the latest IUCN species assessment [40].

All tissue samples were stored at −20°C until analysis. No histopathological examination was performed. Consequently, no health information is available.

### 2.3. Sample Preparation and Analysis

The tissue samples were analyzed for trace elements (Cu, Fe, Mn, Zn, As, Pb, Cd, Hg, Se, Cr, Ni) quantification at the Department of Veterinary Medical Sciences of the University of Bologna following the method described elsewhere [53]. All reagents were from Merck, Darmstadt (Germany); the acids were of Suprapur grade.

Pesticide analysis in the blubber, performed after Covaci et al. [54], included 15 OPs (Dichlorvos, Ethyl paraoxon, Methyl parathion, Disulfoton, Ethion, Azinphos-methyl, Cis-chlorfenvisphos, Diazinon, Fenitrohion, Chlorpyrifos, Fenthion, Malathion, Parathion, Profenofos, Trifluralin) and 15 OCs and metabolites (alpha-lindane, lindane, delta-lindane, heptachlor, aldrin, epoxyheptachlor, dieldrin, endrin aldehyde, endrin ketone, methoxychlor, endrin, endosulfan, 4,4′-DDD, 4,4′-DDE, 4,4′-DDT). Pesticide analysis was gas-chromatographically performed in the Amin Danesh-pazhohan laboratory with an AGILENT-MS5975C, MODE EI GC 7890N equipped with an MS detector [55,56].

### 2.4. Quality Assurance and Quality Control

To check for chemical purity during the trace elements analysis, two blanks were run during each set of analyses, and the accuracy of the method was verified using reference materials (DORM-4 fish protein, National Research Council of Canada). Trace element concentrations in the blubber, liver, and kidney are expressed as mg/kg wet weight.

For pesticide analysis, the extraction, clean-up, and analysis procedures were checked through the regular analysis of procedural blanks and the recovery monitoring of spiked samples, certified material CRM 350 (PCBs and OCPs in mackerel oil), and ERMBC403 (various pesticides in cucumber, which is, at present, the only certified reference material existing for OPs).

Data on the QA-QC results are reported in Table 1. All the values of the reference materials were within certified limits.

### 2.5. Statistics

Data were initially checked for normality by performing a Shapiro–Wilk test. As the data were not normally distributed, non-parametric statistics were used. A Mann–Whitney U-test was used to compare the mean contaminants values between the areas, age classes (juvenile vs. adult), and sex. The Kruskal–Wallis test, followed by posthoc analysis, was used to compare trace element concentrations in the tissues. No area-dependent difference was observed, so all data were considered a single pool of animals. In addition, a Spearman’s rank correlation test was used to examine whether a relationship with biometrics existed.

The significance level was set at *p* = 0.05, and all analyses were conducted with STATISTICA 6.0 (StatSoft Italia S.r.l.).

## 3. Results

### 3.1. Biometrics and Age/Sex Composition of the Sample

The biometrics, age, and sex of the seals are reported in Table 2. The reduced number of seals collected (20) was because we had to rely on fishermen who had to land by-catched animals. Additionally, we should consider that the species is endangered, so we could not perform a specific collection of seals.

### 3.2. Concentrations of Trace Elements in Caspian Seals Tissues

The analytical results of the Caspian seal’s blubber, liver, kidney, heart, and muscle are reported in Table 3 on a w.w. basis. As expected, the highest concentrations were found for Fe in all tissues, while As was among the trace elements presenting the lowest concentrations in all the tissues. The liver showed the highest concentration of all elements, but Cr, Ni, and Cd (highest in the kidney) and blubber had the lowest concentration for all contaminants.

No correlation of Se with hepatic Hg was found, despite the high level of Hg detected.

### 3.3. Concentrations of Pesticides in Caspian Seals Blubber Tissues

**Figure 2:**
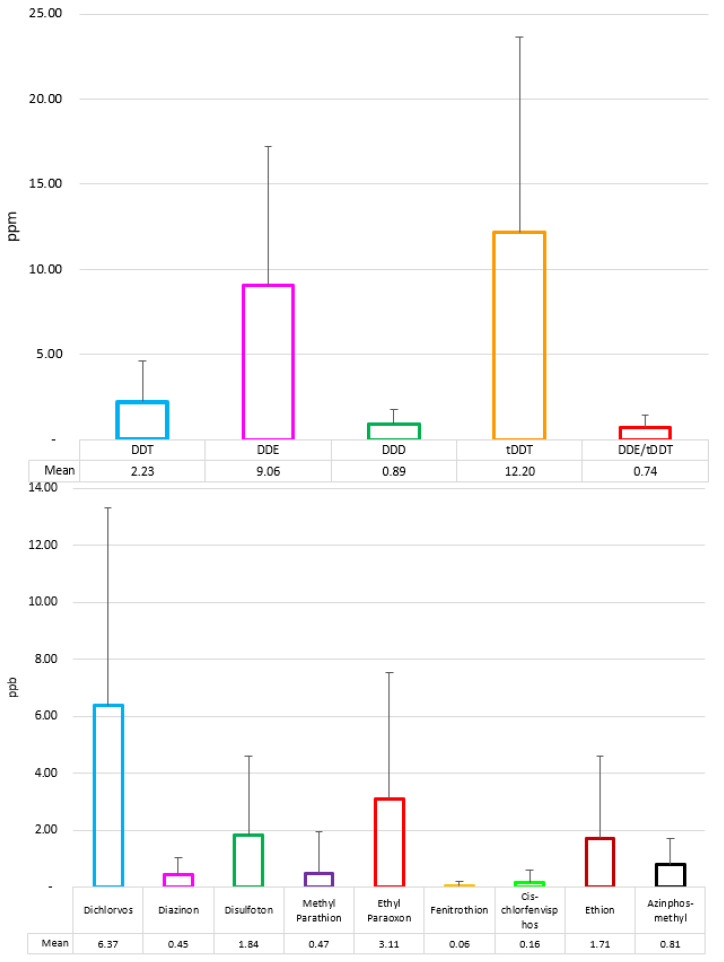
Average concentration on a lipid weight base of DDTs (ppm) and OPs (ppb) in the blubber of south-eastern Caspian seals. No other OCs were detected in the samples.

Out of the 14 OCs analyzed in the present study, only DDTs were detected in all of the samples (Figure 2).

OPs were detected in the present study at a much lower concentration than DDTs (Figure 2).

### 3.4. Influence of Age and Sex on Tissue Distribution

**Figure 3:**
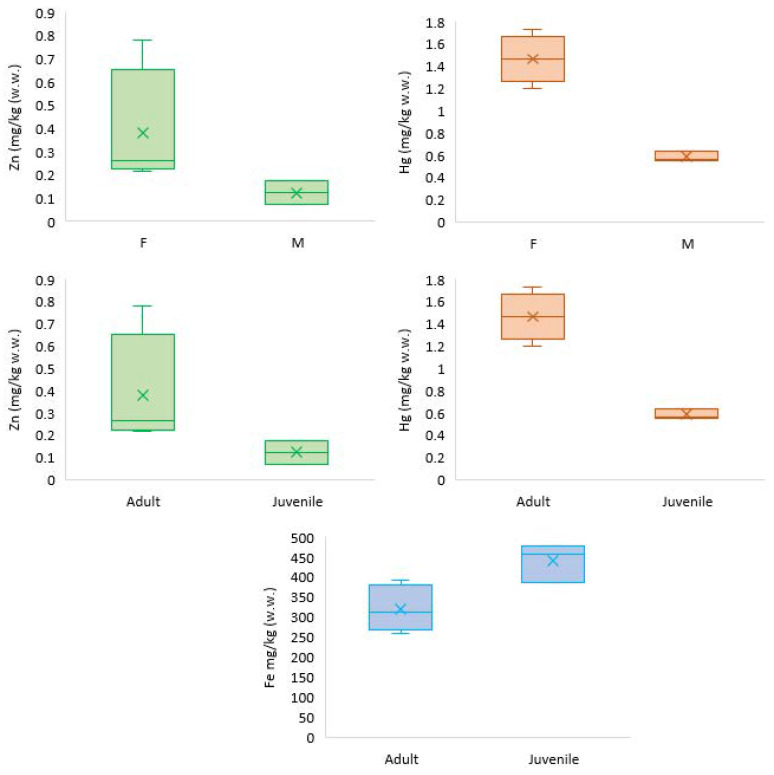
Statistically significant differences were detected in blubber and kidney by age and sex.

In the present study, period- and sex-related variations were detected for Zinc and Hg in the blubber by sex and age and Fe in the kidney by age only. All other trace elements showed no significant variation (Figure 3).

No sex-related difference was found in any of the tissues for Fe. By contrast, age-related differences were found in the kidney (*p* = 0.038), with juveniles presenting higher levels of Fe with respect to adults (Table 3). This difference is supported by a negative correlation between kidney Fe concentrations and NBFL and NTL (*p* = 0.0414 and *p* = 0.0239, respectively).

Age- and sex-related differences in Zn content were found for blubber only (*p* = 0.034 in both cases), with females and adults showing the highest concentrations.

Age- and sex-related differences were found for Hg in the blubber only (*p* = 0.034 in both cases), with adult females presenting higher concentrations than juveniles. These differences are also confirmed by the positive correlation of Hg in the blubber with SL (*p* = 0.0033).

Finally, even if no age- or sex-related difference was detected for As, a negative correlation was found for muscular As and NTL (*p* = 0.0203).

**Figure 4:**
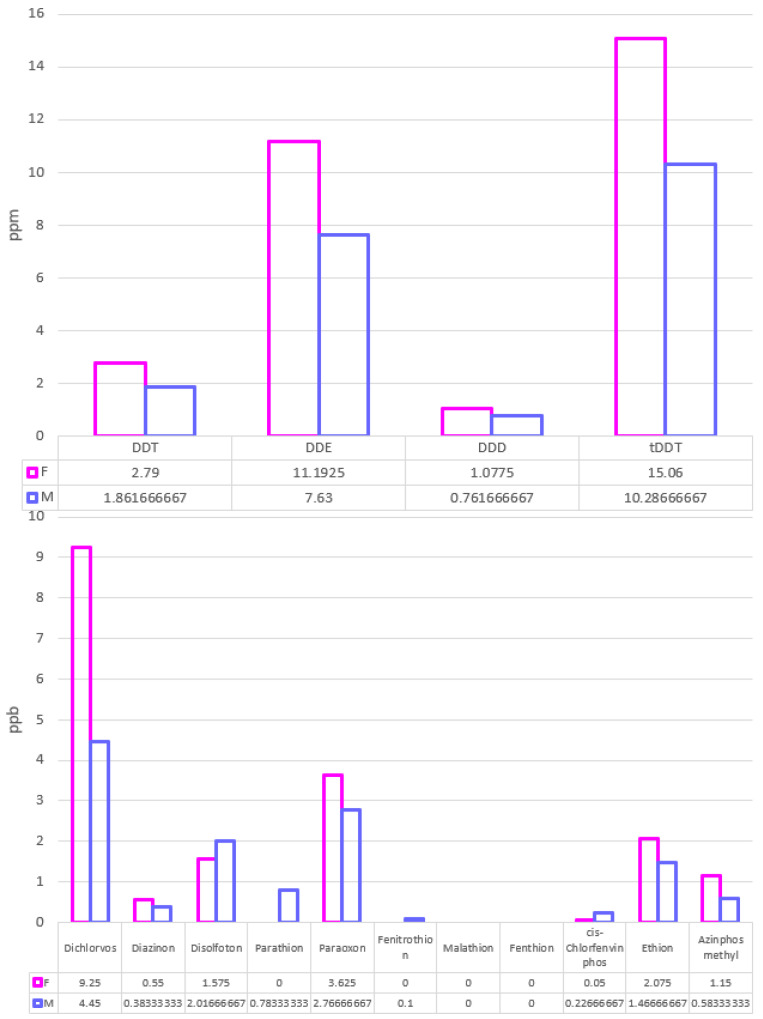
Sex-dependent and average concentration on a lipid weight base of DDTs (ppm) and OPs (ppb) in the blubber of south-eastern Caspian seals.

No sex-related difference was observed for OCs and Ops in the blubber of the sampled Caspian seals. Their concentrations were higher in female seals than in males (Figure 4), but the difference was not significant. Only disulfoton, methyl parathion, fenitrothion, and cis-chlorfenvisphos were higher in males than in females (methyl parathion and fenitrothion were not detected in females).

Pesticide residues (diazinon and DDT family) proved to be positively correlated with the age of animals (*p* = 0.001 for diazinon and *p* < 0.001 for DDT, DDE, DDD, and tDDTs).

## 4. Discussion

### 4.1. Biometrics and Age/Sex Composition of the Sample

The sampling cohort is unbalanced in terms of the age of the animals. The ecology of the species can partially explain the reduced number of juveniles: Caspian seals give birth in the Northern part of the Caspian Sea, where younger animals are easier to find. The larger number of adult females in this sampling cohort can explain the biometrics apparently contrasting what is expected for the species [26,40], as females are larger than males (133.54 ± 4.61 cm vs. 110.95 ± 20.13 cm). When analyzing age/sex composition in detail, all the juveniles were males, so they were smaller than adult females, which can explain this discrepancy. Females also have a thicker blubber layer, but age differences can partially explain this difference.

### 4.2. Concentrations of Trace Elements in Caspian Seals Tissues

Compared with previous research on Caspian seals (Table 4), almost all toxic elements show lower or comparable mean concentrations, except for Pb, which presents higher levels than those reported in previous studies. A decrease in the tissue concentration was observed for Zn and Se [16,26,57].

The observed Hg concentrations are similar to or slightly higher than those reported for the muscle and kidney in previous studies on Caspian seals and lower than the hepatic values reported for many seal species [16,18,22,57,58,59,60,61,62]. Blubber concentrations were higher than those reported for other seal species, i.e., Monk and common seals [22,58,63]. The Caspian seal is considered a Hg-accumulating species, in contrast to the Cd-accumulating species. In this study, Hg and Cd concentrations agree with such a classification. Differential accumulation profiles were used as predictors of prey items: species accumulating high levels of Cd are supposed to feed mainly on invertebrates (i.e., crustaceans, krill, clams, bivalves, cephalopods), which are known to accumulate high amounts of Cd [64]. Species preferentially accumulating Hg are supposed to prey on fish. Thus, the observed Hg/Cd profile confirms the fish preference of Caspian seals [26].

The Se concentrations observed were lower than in previous studies in the liver and kidney and were similar in muscle [16,57]. Selenium concentrations in the liver and kidneys were much lower than those reported for other phocids [16,18,22,58,61,65,66,67]. As already said, no correlation with hepatic Hg was found, despite the high level of Hg detected. Such a correlation is essential because Se has an antagonistic action to the toxic effects of mercury. This detoxifying activity is attained only after the total mercury concentrations have exceeded a specific threshold, which varies from species to species [68]. The antagonistic action of Se to Hg in the liver of different marine mammals has been reported at a molar ratio of Hg: Se of approximately one is usually associated with a high correlation between the two elements.

Levels of Zn in the present study were lower when compared with those reported by [16,26,57] in Northern Caspian seals. The normal range of Zn in marine mammals is 20–100 mg/kg w.w. in the liver [69]. The whole cohort’s hepatic mean value fell inside this range, but females and adults were below the minimum limit of the range. Consequently, this meant that hepatic concentrations raised concern regarding Zn deficiency, impairing the immune system and reproductive activity [70,71,72,73,74].

Compared with data from the other two papers relating to Fe concentrations in Caspian seal tissues, the mean levels detected in all tissues in the present study were higher than those reported by Watanabe et al. [26] for seals caught in 1993 and by Anan et al. [16] in the kidney of dead seals following a distemper virus outbreak in 2000; on the contrary, the hepatic and muscular concentrations of seals in 2000 were higher than those observed in the present study. The origin of seals might partially explain these differences, as previous studies sampled seals from the Northern part of the Caspian Sea. By contrast, animals in the present study were found in the Southern part of the Sea. Although seals should be considered part of the same population, a differential exposure to trace elements should be regarded as, as proved by comparing the metal content in natural prey [26,75].

Caspian seals present the same levels of hepatic Cu as those reported in other species belonging to the subgenus *Pusa* and lower concentrations with respect to those reported for seal species belonging to the other seal genus [26]. After these authors, there was a positive correlation between body size and hepatic Cu; subgenus *Pusa*, with a shorter body length, shows the lowest concentrations consequently. A previous study reported that animals with CDV presenting the highest hepatic Zn had lower Cu concentrations. Similar results have been observed in the present research, associating a negative correlation between Cu and Fe (*p* = 0.5393). It could then be hypothesized that the highest exposure to Zn and Fe combined could have reduced Cu’s absorption [16,76]. 

Compared with the existing studies, Mn hepatic concentrations in the present study are consistently lower, while renal and muscular concentrations are higher or comparable [26].

Tissues Pb are higher than those reported for the same species in previous studies [16,26,57]. Pb concentrations in Caspian seal blubber (0.03 ± 0.02 mg/kg w.w.) are close to those found in ringed seals from Finland (0.05 mg/kg w.w.) but much lower than those reported for Mediterranean monk seals, harp seals, and grey seals [58,77,78]. Pb concentrations in the liver and kidney in the present study were also higher than those reported for common, ringed, gray, hooded (*Cristophora cristata*), and harp seals (*Pagophilus groenlandicus*) [18,21,22,60,61]. 

Analyzing the data set, some individuals with very high trace element concentrations (outliers) were identified (Table 5). All seals (5) were males, and one only was a juvenile (#9).

Although presenting concentrations between two and six times higher than the mean concentrations (Table 3), most of the identified samples do not raise concern in terms of overt toxicity. On the contrary, three males presented Hg concentrations exceeding the threshold of 16 mg/kg w.w. for the subclinical toxicity for marine mammals suggested by the Arctic Monitoring and Assessment Program [79]. These seals represent 15% of the sampling cohort. They are to be considered a warning indication concerning Hg contamination, which should be regarded as a risk for Caspian seals from Iran. Additionally, it should be acknowledged that chronic exposures to even low mercury concentrations have led to enzymatic and hormonal responses, changes in behavior and sensory functions, a reduction in appetite, and, consequently, weight loss in various mammals [12,80,81]. Therefore, it is of the utmost importance to constantly monitor mercury exposure in this endangered species.

Finally, it is worth noting juvenile #9: despite being the youngest of the five males, he presents notably the highest levels of many trace elements, which might indicate a specific point exposure to these contaminants or hepatic and renal failure, leading to the reduced excretion of metals. Unfortunately, no data on health status are available, so no concrete hypothesis can be advanced for these observations.

### 4.3. Concentrations of Pesticides in Caspian Seals Blubber Tissues

No information is currently available concerning recent pesticide exposure in the southern Caspian seal sub-population. During a mass mortality event of Caspian seals that occurred in the period 1997–2001, some studies on OCs bioaccumulation in Caspian seals were performed, showing OC levels to be comparable with residue concentration which has been reported to cause immune suppression in other species [39,42,44].

As shown in Figure 2, only DDTs were detected in all samples out of the 14 OCs analyzed in the present study. Some OCs, such as dieldrin, documented in CDV-infected Caspian seals, were not detected in the current study [39]. Dieldrin residues have been recently reported at low concentrations in Caspian fish around the Iranian coast, proving their current use [82,83,84]. The presence of Caspian seals along the Iranian coasts for a reduced time during the year can explain why no sample was positive for dieldrin [85].

Compared with previous studies on Caspian seals, the detected concentration of tDDTs in the current study was in the lower range [39,41,45,86], which is in agreement with the 2.7-fold decline reported by [87] during 1992–1998. Study [88] reported that a DDE/tDDT ratio lower than 0.6 indicates past exposure to DDT, while higher values are markers of recent exposure. In the present research, a ratio of 0.7 was found, making it possible to suspect a recent exposure of Caspian seals to DDT. Nevertheless, Hall et al. [41] reported no recent exposure to DDTs in Caspian seals from the Northern Caspian Sea in 1993. Recent surveys on some species of Caspian fish around the Iranian coasts revealed new inputs of DDTs into the Caspian Sea [89]. This, together with the DDE/tDDTs ratio reported in Caspian seals (present study), makes it possible to consider an extensive past use of DDTs and illegal use around the Caspian Sea.

Many factors can affect the concentration pattern of detected OPs in Caspian seals [90,91]. For instance, given the low environmental persistence of OPs, their detection in blubber provides evidence for a large environmental presence of these pesticides around the Caspian Sea [35]. Many OPs have been detected in Iran’s Caspian Sea fish and rivers, sometimes exceeding the allowed limits for water and fish, although Caspian seals present a different residual profile [90,92,93].

Surprisingly, to the best of our knowledge, there is no other study on OP exposure in marine mammals to compare the results from the present study. However, detecting OPs in Caspian seals underlines the necessity of performing such studies on other marine mammal species to understand the possible threats from these pesticides on marine ecosystems.

### 4.4. Influence of Age and Sex on Tissue Distribution

Age-related trends and correlations in trace elements have already been reported in pinnipeds and other marine mammals [57,58,94,95,96,97,98,99]. In contrast, marine mammals usually do not show sex-related differences in trace element concentrations except in Hg [58,94,96,97,100,101].

Age-related differences in Fe concentrations (Table 3) are in contrast with what was reported by Watanabe et al. [26], who found higher levels of Fe in the liver of adult females with respect to juveniles (pooled sexes) and adult males and comparable concentrations between ages and sex in the kidney. Additionally, this contrasts with what was stated by Watanabe et al. [102] in the Baikal seal and by Watanabe et al. [26] in the Caspian seal, reporting that Fe burdens increase with age and with diving capacity. So, for example, the Baikal seal has higher muscular Fe levels with respect to the Caspian seal, which has a smaller diving capacity. Additionally, the adults of both species present higher Fe levels with respect to juveniles, and concentrations increase as the diving capacity improves with age. Differing from what was reported by Watanabe et al. [26], in the present study, females showed a smaller variation in hepatic Fe with respect to males (Table 3). Due to reproductive activity, Watanabe et al. [26] ascribe this greater variability to a higher Fe request by females. Indeed, starting from the ecology of the species [40], it could be hypothesized that females from the Northern Caspian Sea were actively reproducing and pregnant (as this area of the basin is the primary reproductive area). Thus, their Fe request increased. The authors suggest that pregnant females showed lower hepatic Fe and Zn concentrations due to a mother-fetus transfer of essential elements. Females from the present study were outside the reproductive period. Consequently, their Fe request /loss was reduced.

Similar to Fe, hepatic concentrations of Zn apparently contrast with those previously reported for Caspian and Baikal seals [26,102], as males and juveniles presented higher levels than females (Table 3).

Age- and sex-related differences for Hg agree with what was reported in the literature. Female seals are known to accumulate higher mercury concentrations than males [58,63,103,104,105,106]; these sex-related differences are believed to be linked to the hyperactive metabolism of female seals and the differences in reproduction and food intake [62,103,107]. Various marine mammals have reported Hg age-related increases [57,108,109] due to the high affinity of Hg to the -SH groups of tissue proteins, leading to a continuous accumulation of this trace element [57,110]. In the case of blubber, most of the Hg in living organisms is in the form of methyl mercury (MeHg), a highly lipophilic compound, easing its accumulation in lipid-rich tissues. Indeed, MeHg is the only metallic compound for which biomagnification is known [111,112,113,114].

No sex-related differences were observed for OCs and OPs in the blubber of sampled Caspian seals, although their concentrations were higher in female seals than in males. Hall et al. [41], Watanabe et al. [45], and Kajiwara et al. [39] also reported no sex-related difference in OCs concentration in Caspian seals. Nevertheless, Tanabe et al. [87] reported higher DDT residues in the blubber of males (17 ppm lipid w.w.) than in females (10 ppm lipid w.w) in Caspian seals sampled in 1998 [39,41,45]. Such a trend has been recorded in other species of marine mammals, and it is well-recognized that lipophilic pesticides decrease in females through lactation and breeding [115,116]. In the present study, an opposite trend was observed. Females were slightly older than males (18.4 ± 5.4 vs. 14.7 ± 7.7 years, respectively), and this age difference was not high enough to be considered a possible explanation for the trend observed. Reproduction failure and lower periods of lactation in Caspian seals can partially explain the differences in pesticide concentration in males and females observed in this and other similar studies on Caspian seals [39,45,85,117]. It should also be remembered that Caspian seals present a reproductive rate of about 0.5, which is considered low, and attributed by some authors to long-term exposure to OCs [45,118]. Even though the accumulation of contaminants with age has been documented in Caspian seals, most animals (including those in the present study) appear below the thresholds associated with reproductive pathologies [46]. This general, low reproductive rate can partially explain the differences between males and females.

Pesticide residues (diazinon and DDT family) have been proven to be positively correlated with the age of animals (*p* = 0.001 for diazinon and p < 0.001 for DDT, DDE, DDD, and tDDTs). Considering the lipophilicity of these compounds, this is an expected result. Similarly, age has been reported as one of the factors influencing Caspian seal OC concentrations. A higher degree of OCs has been reported in older Caspian seals surveyed by Watanabe et al. [45] and Wilson et al. [46]. Kajiwara et al. [42] did not find any relation between age and OC concentration. Additionally, this age-related trend in OC accumulation has been reported in male harp seals (*Phoca groenlandica*) and hooded seals. Still, a negative association between the OCs concentration and age in females was also reported. The authors explained this result with the elimination of OCs through breeding and lactation [115,119]. An increase in the OC and OP residue in both male and female Caspian seals can be explained by higher exposure to OCs and OPs over a lifetime, the biomagnification of OCs and OPs, and the absence of pregnancy and lactation in four sampled Caspian seals in this study [46].

## 5. Conclusions

This is the first assessment of contaminant exposure in the endangered Caspian seal in the Southern Caspian Sea. Contaminants can have a severe impact on the health of marine mammals, and this is particularly true in the case of the Caspian Sea. This semi-closed basin can consequently accumulate vast amounts of pollutants. 

Even though the assessment of trace element exposure alone, i.e., without additional studies on their sub-chronic effects, can potentially provide incorrect information about their actual impact on a species’ health and their role as a conservation threat, it is essential to determine the levels of exposure, as they contribute to the definition of thresholds that define the physiological and toxic values in animals [120]. 

Trace element concentrations in the tissues of Caspian seals in our study showed a different trace element profile with respect to the ones defined for Northern Caspian seals, both healthy or with CDV [16,57], and in some cases, raise concerns on possible overt toxicity (i.e., Hg, Fe). Our study’s low trace element concentrations are within the ranges known to induce sub-chronic effects. Therefore, some potentially harmful effects of some of the trace elements analyzed could not be ruled out. Consequently, it is essential to continue monitoring trace element concentrations in Southern Caspian seals, focusing on Hg: an important threat to several marine species [121,122,123,124].

Considering the endangered status of the Caspian seal and the potentially adverse effects that trace element exposure can have on it, it is of the utmost importance to integrate the analysis of trace element exposure into the standard monitoring protocol for the species. 

From a health status point of view, detecting DDTs in the Caspian seal, even at a lower concentration than those reported to induce possible pollutant-related effects, is a thoughtful alarm [39,125]. Furthermore, OP detection in this species reflects the dangerous possible adverse effects of these components on the Caspian Sea ecosystem and this endangered species [126]. As mentioned, there is no scientific report on the impacts of OCs and OPs on this species. So, individual systemic surveys in closed pools seem beneficial to put light on the information. Additionally, as pesticides occur in complex mixtures, urgent long-term time series complementary surveys are suggested to obtain data about OCs and OPs threats on the health status of the Caspian Sea.

To reduce such a risk for the environment and the species, it is of great importance to improve and control this situation by educating farmers on the correct use of pesticides in terms of dosage, methods, and the suitable time of application. Special training, lectures, and educational TV programs must be developed and offered to authorities and local communities in the region [126]. It is the authors’ opinion that Environmental Agencies of Countries insisting on the Caspian Sea basin intensify the inspection of inappropriate activities that promote the contamination of the Caspian Sea, punishing those involved by environmental legislation.

Finally, considering the migratory lifestyle of Caspian seals, the creation of protected areas within shores and islands that these species prefer is recommended.

## Figures and Tables

**Table 1:** Summary of QA-QC data for trace elements and pesticide analysis.

Compound	Units	LOD	LOQ	Recovery%
beta-Mevinphos	ppb	0.66	2.20	69
Trifluralin	ppb	0.39	1.30	76
Diazinon	ppb	0.56	1.86	82
Disolfoton	ppb	1.30	4.33	81
Methyl Parathion	ppb	0,88	2.93	70
Ethyl Paraoxon	ppb	0.76	2.53	89
Fenitrothion	ppb	0.78	2.60	87
Malathion	ppb	0.65	2.16	79
Fenthion	ppb	0.12	0.40	88
Chlorpyrifos	ppb	0.19	0.63	79
Parathion	ppb	0.32	1.07	102
Bromofos	ppb	0.39	1.30	89
Trans-Chlorfenvinphos	ppb	0.49	1.63	93
Butachlor	ppb	0.55	1.83	81
Profenophos	ppb	0.36	1.20	77
Ethion	ppb	0.39	1.30	106
Azinphos methyl	ppb	0.50	1.50	93
Aldrin	ppb	1.69	5.63	100
Dieldrin	ppb	2.08	6.94	101
Endrin	ppb	2.26	7.52	104
Endrinketon	ppb	1.76	5,86	98
Endrin aldehyde	ppb	1.21	4.02	105
Lindane	ppb	1.43	4.76	102
4,4-DDT	ppb	1.86	6.21	95
4,4-DDE	ppb	1.23	4.11	96
4,4-DDD	ppb	2.16	7.21	96
Endosulfan	ppb	2.92	9.73	100
ά-HCH	ppb	1.11	3.70	105
delta-HCH	ppb	1.45	4.84	96
Heptachlor	ppb	8.95	29.82	98
Heptachlor epoxide	ppb	2.16	7.21	104
Methoxychlor	ppb	4.23	14.11	95
Cu	ppb	0.77	0.95	95
Fe	ppb	0.55	0.73	100
Mn	ppb	0.14	0.23	98
Zn	ppb	0.64	0.83	90
Cr	ppb	0.71	0.81	105
Ni	ppb	1.8	2.03	98
As	ppb	0.1	0.15	112
Pb	ppb	0.1	0.13	96
Cd	ppb	1.8	2.11	99
Se	ppb	0.1	0.15	115
Hg	ppb	0.061	0.10	120

**Table 2:** Summary of samples collected from 20 deceased Caspian seals in Iran (2013–2016). The age of seals is also reported, as determined after Amano et al. [52]. BT: Blubber thickness; SL: Straight length; NTL: AG: Axillary girth.

Seal	Age	Sex	BT (cm)	SL (cm)	AG (cm)
1	18	F	5	131	84
2	17	F	6.1	130	89
3	16	M	5	118	83
4	26	F	5.5	140	88
5	19	F	4.2	132	74
6	27	F	5.3	148	101
7	10	M	0.8	99	75
8	20	M	2.3	136	52
9	6	M	6	95	82
10	19	F	6.5	131	100
11	11	F	4.2	115	104
12	11	F	2.8	125	65
13	25	M	6	149	95
14	9	M	5.9	98	83
15	15	F	4.8	128	100
16	10	M	4.8	100	90
17	21	F	4.6	145	97
18	7	M	3.9	96	82
19	12	M	5.5	120	96
20	28	M	7	150	99

**Table 3:** Concentrations of trace elements in Caspian seal tissues collected from 2013 to 2016 in Iran, expressed as M ± SD (mg/kg w.w.). The total number of samples for all tissues was 20:10 from females and 10 from males.

**Tissue**		**Cu**	**Fe**	**Mn**	**Zn**	**Cr**	**Ni**
Blubber	Total	0.62 ± 0.33	8.86 ± 5.80	0.14 ± 0.03	0.27 ± 0.24	0.16 ± 0.11	0.09 ± 0.08
	Females	0.58 ± 0.32	9.85 ± 7.55	0.15 ± 0.03	0.38 ± 0.27	0.15 ± 0.09	0.10 ± 0.11
	Males	0.69 ± 0.39	7.53 ± 3.27	0.12 ± 0.03	0.12 ± 0.05	0.18 ± 0.16	0.07 ± 0.02
Liver	Total	13.19 ± 5.40	543.50 ± 570.93	2.95 ± 1.85	30.1 ± 47.30	0.29 ± 0.17	0.13 ± 0.05
	Females	15.17 ± 3.35	372.57 ± 136.39	2.17 ± 1.27	16.99 ± 5.89	0.30 ± 0.12	0.16 ± 0.08
	Males	11.87 ± 6.37	657.45 ± 732.55	3.47 ± 2.10	38.69 ± 61.48	0.28 ± 0.20	0.21 ± 0.13
Kidney	Total	12.88 ± 5.79	360.38 ± 79.89	1.68 ± 1.87	24.82 ± 27.73	0.34 ± 0.13	0.13 ± 0.07
	Females	15.18 ± 4.90	338.33 ± 62.93	1.21 ± 0.56	16.53 ± 4.42	0.33 ± 0.16	0.10 ± 0.03
	Males	11.73 ± 6.27	371.40 ± 90.50	1.92 ± 2.29	28.96 ± 34.06	0.43 ± 0.13	0.14 ± 0.09
Heart	Total	13.21 ± 2.47	207.93 ± 51.78	0.49 ± 0.11	22.65 ± 4.82	0.28 ± 0.11	0.08 ± 0.03
	Females	14.00 ± 0.76	226.86 ± 73.45	0.59 ± 0.11	23.02 ± 0.36	0.36 ± 0.18	0.11 ± 0.05
	Males	12.81 ± 3.05	198.47 ± 48.09	0.44 ± 0.08	22.46 ± 6.21	0.24 ± 0.05	0.07 ± 0.02
Muscle	Total	9.99 ± 9.03	290.46 ± 250.35	2.03 ± 2.26	12.19 ± 9.29	0.33 ± 0.09	0.12 ± 0. 80
	Females	10.57 ± 8.34	301.24 ± 189.71	2.41 ± 1.71	10.41 ± 8.27	0.41 ± 0.11	0.11 ± 0.07
	Males	9.41 ± 9.71	279.68 ± 310.98	1.65 ± 2.81	13.97 ± 10.31	0.24 ± 0.06	0.13 ± 0.09
**Tissue**		**As**	**Pb**	**Cd**	**Se**	**Hg**	
Blubber	Total	0.10 ± 0.05	0.03 ± 0.02	0.02 ± 0.004	0.06 ± 0.06	1.09 ± 0.49	
	Females	0.12 ± 0.05	0.03 ± 0.02	0.03 ± 0.002	0.05 ± 0.05	1.46 ± 0.22	
	Males	0.07 ± 0.04	0.02 ± 0.01	0.02 ± 0.01	0.07 ± 0.07	0.59 ± 0.05	
Liver	Total	0.06 ± 0.03	1.76 ± 4.79	0.61 ± 0.87	0.75 ± 0.44	15.12 ± 15.84	
	Females	0.06 ± 0.04	0.52 ± 0.75	0.77 ± 0.56	0.83 ± 0.56	5.09 ± 2.55	
	Males	0.05 ± 0.02	2.58 ± 6.23	0.51 ± 1.07	0.70 ± 0.39	21.80 ± 17.70	
Kidney	Total	0.06 ± 0.01	0.18 ± 0.28	5.89 ± 14.37	0.29 ± 0.20	5.09 ± 3.72	
	Females	0.05 ± 0.01	0.08 ± 0.06	1.61 ± 1.08	0.28 ± 0.42	4.02 ± 2.12	
	Males	0.07± 0.01	0.22 ± 0.35	8.03 ± 17.70	0.30 ± 0.20	5.63 ± 4.39	
Heart	Total	0.05 ± 0.03	0.07 ± 0.09	0.03 ± 0.01	0.34 ± 0.07	2.79 ± 1.54	
	Females	0.06 ± 0.04	0.02 ± 0.001	0.03 ± 0.01	0.39 ± 0.05	3.08 ± 2.58	
	Males	0.04 ± 0.03	0.09 ± 0.11	0.02 ± 0.01	0.31 ± 0.07	2.65 ± 1.29	
Muscle	Total	0.06 ± 0.02	0.60 ± 1.01	0.18 ± 0.37	0.45 ± 0.55	7.67 ± 2.29	
	Females	0.07 ± 0.02	0.57 ± 0.99	0.15 ± 0.41	0.45 ± 0.51	8.05 ± 2.01	
	Males	0.05 ± 0.01	0.62 ± 1.02	0.21 ± 0.33	0.44 ± 0.58	7.29 ± 2.56	

**Table 4:** Comparison of trace element concentrations in Caspian seal tissues collected in the present study and previous research (mg/kg w.w.). Data from Ikemoto and Ershova are expressed as mg/kg d.w.

Trace Element	Tissue Concentration (mg/kg)					Reference
	Liver	Kidney	Muscle	Blubber	Heart	
Fe	543.50 ± 570.93	360.38 ± 79.89	290.46 ± 250.35	8.86 ± 5.80	207.93 ± 51.78	Present study
	470 ± 580	150 ± 42	200 ± 64			[26]
	543.50 ± 570.93	543.50 ± 570.93	543.50 ± 570.93	543.50 ± 570.93	543.50 ± 570.93	[47]
	1100 ± 510 (2000)	200 ± 97 (2000)	410 ± 200 (2000)			[16]
	481.9 ± 15.6	287.2 ± 12.3	338.9 ± 14.3	367 ± 12.9	280.8 ± 11.4	[47]
Mn	2.95 ± 1.85	1.68 ± 1.87	2.03 ± 2.26	0.14 ± 0.03	0.49 ± 0.11	Present study
	5.5 ± 1.3	1 ± 0.2	0.18 ± 0.06			[26]
	6.37 ± 1.94 (1998) 6.15 ± 3.29 (2000)	1.21 ± 0.2 (1998) 1.01 ± 0.27 (2000)	0.2 ± 0.094 (1998) 1.04 ± 1.25 (2000)			[16]
	20.3 ± 6.1	5.03 ± 0.96	0.671 ± 0.361			[57]
	8.6 ± 0.8	1.9 ± 0.4	0.6 ± 0.04	0.2 ± 0.01	1.3 ± 0.3	[47]
Zn	30.1 ± 47.30	24.82 ± 27.73	12.19 ± 9.29	0.27 ± 0.24	22.65 ± 4.82	Present study
	49 ± 15	27 ± 7	30 ± 9			[26]
	70.9 ± 17.4 (1998) 90.7 ± 52 (2000)	47.9 ± 13 (1998) 58.4 ± 30.8 (2000)	41.8± 10.9 (1998) 44.8 ± 13.2 (2000)			[16]
	226 ± 54.9	199 ± 55	141 ± 38			[57]
	109.4 ± 3.4	87.3 ± 3.4	69.8 ± 3.2	42.5 ± 3.3	63.6 ± 2.1	[47]
Cu	13.19 ± 5.40	12.88 ± 5.79	9.99 ± 9.03	0.62 ± 0.33	13.21 ± 2.47	Present study
	11 ± 5	3.3 ± 0.6	1.1 ± 0.2			[26]
	13.4 ± 9.4 (1998) 5.63 ± 3.54 (2000)	4.12 ± 1.38 (1998) 4.66 ± 1.23 (2000)	1.08 ± 0.34 (1998) 1.6 ± 0.46 (2000)			[16]
	42.9 ± 30.3	17.1 ± 5.7	3.63 ± 1.13			[57]
	31.8 ± 1.1	10.8 ± 0.7	5.8 ± 0.2	0.6 ± 0.04	11.8 ± 0.5	[47]
Pb	1.76 ± 4.79	0.18 ± 0.28	0.60 ± 1.01	0.03 ± 0.02	0.07 ± 0.09	Present study
	0.068 ± 0.046	0.078 ± 0.097	0.027 ± 0.028			[26]
	0.002 ± 0.007 (1998) 0.019 ± 0.011 (2000)	0.031 ± 0.084 (1998) 0.005 ± 0.004 (2000)	0.005 ± 0.014 (1998) 0.018 ± 0.027 (2000)			[16]
	0.006 ± 0.024	0.116 ± 0.311	0.018 ± 0.05			[57]
Ni	0.13 ± 0.05	0.13 ± 0.07	0.12 ± 0. 80	0.09± 0.08	0.08 ± 0.03	Present study
	<0.07	0.07 ± 0.039	<0.04			[26]
Cd	0.61 ± 0.87	5.89 ± 14.37	0.18 ± 0.37	0.02 ± 0.004	0.03 ± 0.01	Present study
	1.1 ± 1.7	9.5 ± 11	0.01 ± 0.017			[26]
	0.732 ± 0.593 (1998) 0.929 ± 1.41 (2000)	12.5 ± 11.2 (1998) 6.99 ± 7.81 (2000)	0.016 ± 0.034 (1998) 0.024 ± 0.014 (2000)			[16]
	2.37 ± 1.94	51.4 ± 44.3	0.054± 0.116			[57]
Hg	15.12 ± 15.84	5.09 ± 3.72	7.67 ± 2.29	1.09 ± 0.49	2.79 ± 1.54	Present study
	15 ± 26	1.6 ± 1.3	0.55 ± 0.30			[26]
	27 ± 23 (1998) 5.8 ± 8.4 (2000)	1.9 ± 3.4 (1998) 1.8 ± 3.1 (2000)	0.44 ± 0.24 (1998) 0.48 ± 0.39 (2000)			[16]
	85 ± 74	8.1 ± 15.4	1.5 ± 0.8			[57]
As	0.06 ± 0.03	0.06 ± 0.01	0.06 ± 0.02	0.10 ± 0.05	0.05 ± 0.03	Present study
	0.17 ± 0.09 (2000)	0.16 ± 0.08 (2000)	0.11 ± 0.04 (2000)			[16]
Cr	0.29 ± 0.17	0.34 ± 0.13	0.33 ± 0.09	0.16 ± 0.11	0.28 ± 0.11	Present study
	0.081 ± 0.081 (1998) 0.11 ± 0.061 (2000)	0.076 ± 0.091 (1998) 0.072 ± 0.033 (2000)	0.015 ± 0.031 (1998) 0.073± 0.016 (2000)			[16]
	0.26 ± 0.27	0.32 ± 0.39	0.05 ± 0.1			[57]
Se	0.75 ± 0.44	0.29 ± 0.20	0.45 ± 0.55	0.06 ± 0.06	0.34 ± 0.07	Present study
	19 ± 13 (1998) 5.2 ± 4.4 (2000)	3.6 ± 1.2 (1998) 2.8 ± 0.9 (2000)	0.66 ± 0.19 (1998) 0.62 ± 0.30 (2000)			[16]
	60 ± 42	15 ± 5	2.2 ± 0.6			[57]

**Table 5:** Concentrations of trace elements as mg/kg w.w. in outlier Caspian seals. Data are reported by individual and by tissue.

Seal ID	Age (years)	Gender	Tissue (mg/kg w.w.)			
			Heart	Kidney	Liver	Muscle
*7*	10	Male			Hg: 31.86	Pb: 1.79
*8*	20	Male	Pb: 0.25		Hg: 42.35	
*9*	6	Male		Cd: 44.16	Fe: 2119.06 Ni: 0.45 Pb: 15.30 Zn: 163.79	
*16*	10	Male		Pb: 0.92		
*20*	28	Male			Hg: 38.79	

## Data Availability

The data presented in this study are available on request from the corresponding authors.
